# Real‐World Safety of Vonoprazan in Patients and Symptom Outcomes in Patients With Reflux Esophagitis in China: A Prospective, Non‐Interventional Study (VIEW)

**DOI:** 10.1111/1751-2980.70051

**Published:** 2026-05-21

**Authors:** Ying Lian Xiao, Hui Yang, Yi Xia Lu, Li Yang, Guo Xin Zhang, Guo Liang Ye, Qi Song, Li Xie, Min Hu Chen

**Affiliations:** ^1^ Department of Gastroenterology and Hepatology The First Affiliated Hospital of Sun Yat‐sen University Guangzhou Guangdong Province China; ^2^ Department of Gastroenterology The Second Affiliated Hospital of Guangzhou Medical University Guangzhou Guangdong Province China; ^3^ Department of Gastroenterology Heilongjiang Provincial Hospital Harbin Heilongjiang Province China; ^4^ Department of Gastroenterology West China Hospital, Sichuan University Chengdu Sichuan Province China; ^5^ Department of Gastroenterology The First Affiliated Hospital of Nanjing Medical University Nanjing Jiangsu Province China; ^6^ Department of Gastroenterology The First Affiliated Hospital of Ningbo University Ningbo Zhejiang Province China; ^7^ China Medical Team, Takeda (China) International Trade Co., Ltd. Shanghai China

**Keywords:** esophagitis, peptic, gastroesophageal reflux disease, potassium competitive acid blockers, vonoprazan

## Abstract

**Objectives:**

Vonoprazan was approved in China for reflux esophagitis (RE) in 2019; however, its real‐world safety and effectiveness remain undetermined. We aimed to evaluate the safety of vonoprazan in patients and its effectiveness in patients with RE in a real‐world setting.

**Methods:**

In this prospective, non‐interventional, real‐world study, vonoprazan 20 mg was administered orally daily for 4 or 8 weeks. Primary endpoints were incidence rates of adverse events (AEs), serious AEs (SAEs), and adverse drug reactions (ADRs). Secondary endpoints included proportions of patients with RE without typical gastroesophageal reflux disease (GERD) symptoms (complete symptom relief) at baseline and Week 4, relief of heartburn/regurgitation symptoms (all day/nighttime) during Week 1, and mean change in GERD Questionnaire (GERDQ) score at Week 4.

**Results:**

Of the 2829 patients with safety data, 488 (17.2%) reported ≥ 1 AE; 29 (1.0%) reported ≥ 1 SAE; and 129 (4.6%) reported ≥ 1 ADR. Of 1796 patients with RE and effectiveness data, the proportion without typical symptoms of GERD increased from 20.1% (95% confidence interval [CI] 18.29–22.05) at baseline to 55.2% (95% CI 52.63–57.79) at Week 4 (change: 35.1%, 95% CI 31.95–38.25). Complete relief from heartburn, nighttime heartburn, regurgitation, and nighttime regurgitation was observed in 32.3% (314/971), 43.8% (427/975), 27.0% (262/971), and 40.5% (395/976) patients, respectively, during Week 1. Mean change in GERDQ score (*n* = 1464) improved by Week 4 (−1.70).

**Conclusion:**

Vonoprazan is well tolerated through 8 weeks in patients and improves symptoms in patients with RE in the real‐world setting in China (VIEW; NCT04501627).

## Graphical abstract

The multicenter, prospective, non‐interventional, real‐world VIEW study of 2829 patients in China treated with vonoprazan found the treatment to be well tolerated, with 17.2% of patients reporting at least one adverse event. Among the 1796 patients with reflux esophagitis (RE), the proportion of those without gastroesophageal reflux disease increased from 20.1% at baseline to 55.2% at Week 4 of treatment, indicating that patients with RE receiving vonoprazan experienced effective symptom relief in a real‐world setting.

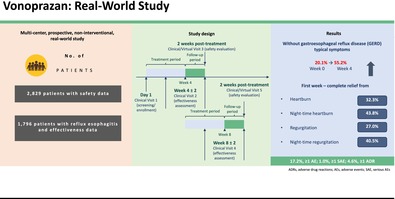

## 1 Introduction

Reflux esophagitis (RE) is a common type of gastroesophageal reflux disease (GERD) characterized by mucosal breaks and/or typical or atypical symptoms [1, 2]. Typical symptoms of RE include heartburn and regurgitation, whereas atypical symptoms consist of noncardiac chest pain, cough, and asthma, among others [2, 3, 4, 5, 6, 7]. These symptoms may affect daily functions of the patients and impair their quality of life (QoL), leading to a significant economic burden on the healthcare system [8, 9].

In China, the prevalence of endoscopically diagnosed RE has been reported to be 6.4% in 2011 [10]. The treatment goals for patients with RE are to alleviate symptoms, heal esophagitis, improve QoL, avoid symptom recurrence, and avert or treat complications [11, 12, 13]. Proton pump inhibitors (PPIs) have been a mainstay therapy for patients with RE [14, 15]. However, their efficacy for treating RE is now considered to be limited, given their short half‐life (approximately 1–2 h), requiring several doses to achieve maximum acid suppression and symptom relief. In addition, high intra‐ and inter‐individual variation in pharmacokinetics may also influence their treatment effectiveness [16, 17, 18]. Some patients with RE experience low rates of mucosal healing and symptom relief after PPI administration, as well as recurrence of esophagitis [19].

Vonoprazan is a novel oral potassium‐competitive acid blocker (P‐CAB), which suppresses acid secretion by binding to both active and inactive proton pumps [13, 20]. Unlike PPIs, vonoprazan provides potent and prolonged gastric acid suppression, given its long half‐life of 7–9 h and p*K*_a_ value of 9.06, a rapid onset of action from the first dose, lack of cytochrome P450 2C19 (CYP2C19)‐based interactions, and convenience of administration (e.g., not dependent on mealtimes) [21, 22, 23, 24, 25, 26, 27]. Vonoprazan has been approved in Japan indicated for gastrointestinal (GI) diseases such as gastric ulcer, duodenal ulcer, and RE; recurrent gastric or duodenal ulcer associated with low‐dose aspirin or non‐steroidal anti‐inflammatory drugs; and as an adjunct therapy for *Helicobacter pylori* eradication (see Supporting Information) [28]. In the United States, vonoprazan has been approved for indications including the healing and maintenance of RE in adults and in combination therapy for *H. pylori* eradication in adults (Supporting Information) [24]. In China, it has also been approved for treating RE and as an adjunct therapy for *H. pylori* eradication [29, 30, 31]. In addition, two phase III trials that included patients in China have demonstrated noninferior efficacy and safety of vonoprazan in RE or healed RE to prevent disease recurrence when compared with PPIs [11, 32]. However, as with all newly approved medications, patients are served by continuing real‐world pharmacovigilance assessment. Also, the safety profiles of all newly approved medicines need to be intensively monitored in a real‐world setting. Furthermore, there is a scarcity of reports on the real‐world safety and effectiveness of vonoprazan in patients with RE in China.

Therefore, we conducted this prospective, observational, real‐world study to evaluate the safety profile in patients receiving vonoprazan and to assess its effectiveness in those with RE in China.

## 2 Patients and Methods

### 2.1 Study Design and Patient Enrollment

VIEW is a real‐world, multicenter, single‐arm, prospective, non‐interventional study (registration no. NCT04501627) that was conducted in 39 institutions across China (Table S1). The study investigated the safety profile (primary endpoint) and both treatment effectiveness (secondary endpoint) and QoL (exploratory endpoint) in patients with RE who were treated with vonoprazan between December 2020 and May 2022. Diagnosis of RE was made based on Medical Dictionary for Regulatory Activities (MedDRA) version 23.1 Lowest Level Terms (LLTs) coded as “reflux esophagitis,” “erosive esophagitis,” “reflux oesophagitis,” “erosive oesophagitis,” or “ongoing.” Adult (aged ≥ 18 years) patients with RE who were receiving vonoprazan and provided signed informed consent indicating that they (or their legal representative) were fully informed of the study and were willing to participate were included in the study. Exclusion criteria included: (i) patients who were currently enrolled in or planned to participate in another clinical trial (i.e., an interventional study); and (ii) patients who were contraindicated for vonoprazan per the Product Package Insert [24]. All patients were given oral administration of vonoprazan 20 mg daily for 4 weeks, which was prolonged to 8 weeks if there was insufficient benefit, as informed by local prescribing information [33], and was followed by a safety follow‐up at 2 weeks post‐treatment (Figure S1). Prior or concomitant medications were permitted. All treatment decisions were made at the investigator's discretion, including those related to dose adjustment and treatment duration, as well as the method and frequency of clinical assessments (Supporting Information).

The study was conducted in compliance with the Declaration of Helsinki, the International Society for Pharmacoepidemiology Guidelines for Good Epidemiology Practices, European Network of Centers for Pharmacoepidemiology and Pharmacovigilance Guidelines for Methodological Standards in Pharmacoepidemiology, Good Pharmacovigilance Practices, and all applicable regulatory requirements. Each center complied with the regulatory requirements in China, and the study was approved by all centers before initiation. This study was approved by the Institutional Ethics Committee for Clinical Research and Animal Trials of the First Affiliated Hospital of Sun Yat‐sen University (no. [2020] 228), and the study protocol was reviewed and approved by the Ethics Committee at each participating institution. All patients (or their legal representative) provided written informed consent before enrollment.

### 2.2 Study Endpoints

The primary endpoints were incidence rates of adverse events (AEs), serious AEs (SAEs), and adverse drug reactions (ADRs). AEs were coded using the MedDRA Version 23.1 (issued on September 1, 2020) and were defined as any untoward medical occurrence in a patient administered a medicinal product, which does not necessarily have a causal relationship with this medicinal product. SAEs were defined as events recorded as “serious” on the “Adverse Event Details” page of the clinical report form. ADRs were defined as AEs related to vonoprazan.

The secondary endpoints included the proportion of patients with RE in the absence of typical GERD symptoms at baseline and Week 4. Additional secondary endpoints were the proportions of patients with RE who had heartburn all day, nighttime heartburn, regurgitation all day, or nighttime regurgitation at baseline who achieved complete symptom relief of heartburn, nighttime heartburn, regurgitation, or nighttime regurgitation from Day 1 to Day 7 of the first week and for ≥ 7 consecutive days between Day 1 and Day 14 of the first 2 weeks based on the patient's symptom diary (Table S2). Further secondary endpoints were the change in GERDQ score [34] from baseline to Week 4 in patients with RE, and the endoscopic healing rate—calculated as the number of patients with healed RE divided by that of healed and unhealed RE cases—at Week 4 and Week 8 in patients with RE who had no mucosal breaks under endoscopy. Exploratory endpoints included the following in patients with RE: (i) improvement in QoL scores from baseline to Week 4 for the Pittsburgh Sleep Quality Index (PSQI) [35]; and (ii) the EuroQol 5‐Dimension 5‐Level (EQ‐5D‐5L) [36] and EuroQol‐Visual Analogue Scale (EQ‐VAS) [37].

### 2.3 Statistical Analysis

The target number of patients to register was set as 3000. This sample size was determined based on a “rule of thumb” that for ≥ 3000 patients, even for AEs occurring at a frequency of 1 in 1000, occurrence of at least one of such events would likely be observed. This sample size also provided sufficient precision for detecting symptom improvement endpoints (e.g., the proportion of patients with RE in the absence of typical GERD symptoms at baseline and Week 4, relief of heartburn/regurgitation symptoms [all day/nighttime] in the first week, and mean change in GERDQ score at Week 4). Descriptive statistics were conducted for categorical and numerical variables (Supporting Information). Safety analyses were conducted in the safety analysis population (SAP) who received at least one dose of investigator‐prescribed vonoprazan in routine care and provided safety information. Effectiveness analysis was conducted in the effectiveness analysis population with reflux esophagitis (EAPRE), which was defined as patients in the SAP who had at least one effectiveness endpoint assessment at Clinical Visit 2 (Week 4 ± 2) and/or Clinical Visit 4 (Week 8 ± 2), and with MedDRA LLTs coded as “reflux esophagitis,” “erosive esophagitis,” “reflux oesophagitis,” “erosive oesophagitis,” or “ongoing.” Imputation was not applied in cases where effectiveness data was missing or a patient discontinued from the study. For subgroup analyses of the EAPRE, descriptive statistics were conducted for secondary and exploratory endpoints, including patients who had RE with Los Angeles classification grade (hereafter referred to as LA grade) A/B or C/D. Unless otherwise specified in the description of the analyses, a two‐sided 95% confidence interval (CI) was considered as a default (two‐sided significant level [*α*] 5% [< 0.05]). Negative values represented improvement for GERDQ and PSQI scores, and positive values represent improvement for EQ‐5D‐5L and EQ‐VAS scores.

## 3 Results

### 3.1 Patient Disposition

**FIGURE 1:**
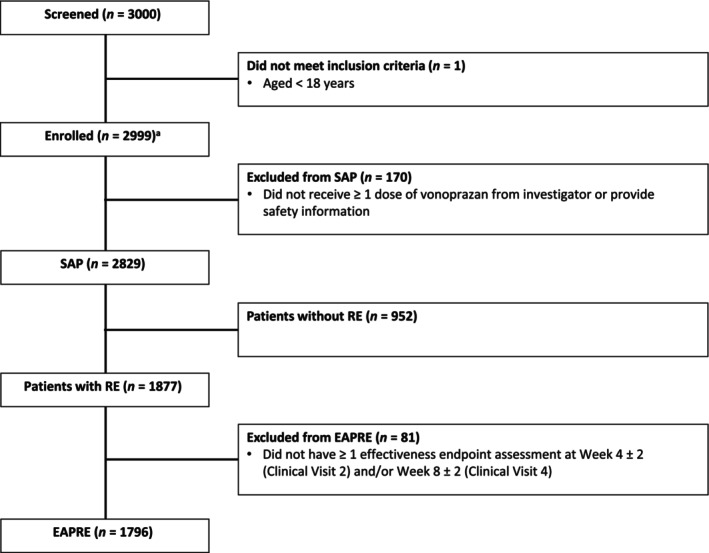
Patient disposition and analysis populations. ^a^Patients discontinued the study (*n* = 356) due to adverse events (AEs) (*n* = 26), physician decision (*n* = 3), lost to follow‐up (*n* = 121), withdrawal by patient (*n* = 199), not taking medication as scheduled (*n* = 1), or patient not taking medication (*n* = 6). A total of 2643 patients completed the study. EAPRE, effectiveness analysis population with reflux esophagitis; RE, reflux esophagitis; SAP, safety analysis population.

Patient data were collected between December 2020 and May 2022. Following the screening of 3000 patients, a total of 2999 adult patients were enrolled. Of these patients, 356 (11.9%) discontinued the study, with the most common reasons being withdrawal by the patient (*n* = 199, 6.6%) and lost to follow‐up (*n* = 121, 4.0%). The remaining 2643 (88.1%) patients completed the study. The SAP comprised 2829 patients, and the EAPRE included 1796 patients from the SAP. The study flowchart is shown in Figure 1.

At baseline, the mean age of patients in the SAP was (48.3 ± 13.8) years, and 1642 (58.0%) of them were men. RE was diagnosed in 1877 (66.3%) patients (Table 1). In the SAP (*n* = 2829), the mean number of days between the first and last doses of vonoprazan exposure was 31.7 ± 17.9 days. An overview of patients who received prior or concomitant medications is shown in Table S3.

**TABLE 1:** Baseline characteristics of safety analysis population.

Variables	Overall population (*N* = 2829)
Age, years (mean ± SD)	48.3 ± 13.8
Sex (*n*, %)	
Male	1642 (58.0)
Female	1187 (42.0)
BMI, kg/m^2^ (mean ± SD)	23.7 ± 3.5
Missing data (*n*, %)	1 (0.04)
Smoking (*n*, %)	
Current smoker	456 (16.1)
< 20 cigarettes per day	312 (68.4)
20–60 cigarettes per day	138 (30.3)
> 60 cigarettes per day	6 (1.3)
Former smoker	197 (7.0)
Never smoker	2175 (76.9)
Missing data	1 (0.04)
Alcohol consumption (*n*, %)	
Daily drinking	106 (3.7)
Drink several days weekly	178 (6.3)
Drink several days monthly	367 (13.0)
Never	2175 (76.9)
Missing data	3 (0.1)
RE (*n*, %)	
Yes	1877 (66.3)
No	952 (33.7)
Barrett's mucosa (*n*, %)	
Yes	61 (2.2)
No	2672 (94.4)
Missing data	96 (3.4)
Esophageal stricture (*n*, %)	
Yes	47 (1.7)
No	2686 (94.9)
Missing data	96 (3.4)
Esophageal ulceration (*n*, %)	
Yes	260 (9.2)
No	2473 (87.4)
Missing data	96 (3.4)
*H. pylori* status (*n*, %)	
Positive	583 (20.6)
Negative	1102 (39.0)
Unknown	1144 (40.4)

Abbreviations: BMI, body mass index; *H. pylori* , *Helicobacter pylori* ; RE, reflux esophagitis; SD, standard deviation.

### 3.2 Primary Endpoints: AEs, SAEs, and ADRs

Among the 2829 patients in the SAP (Figure 1), 488 (17.2%) experienced at least one AE during the study (Table 2), where most AEs were mild (14.8%) or moderate (3.2%) (severe: 0.6%). The most common AEs were system organ class‐classified GI disorders, which were mild or moderate in 240 (8.5%) patients, and severe in 2 (0.1%) patients, respectively. Of these GI disorders, by preferred terms, diarrhea was most frequent (*n* = 41, 1.4%), followed by nausea (*n* = 37, 1.3%), GERD (*n* = 36, 1.3%), and dyspepsia (*n* = 34, 1.2%). Other common AEs included infections and infestations in 96 (3.4%) patients—most commonly including upper respiratory tract infection (*n* = 19, 0.7%), gastroenteritis (*n* = 12, 0.4%), and pharyngitis (*n* = 8, 0.3%)—as well as nervous system disorders in 60 (2.1%) patients. Moreover, 29 (1.0%) of 2829 patients in the SAP reported at least one SAE, the most common being infections and infestations (*n* = 5, 0.2%).

**TABLE 2:** Adverse events (AEs), serious adverse events (SAEs), and adverse drug reactions (ADRs) in the safety analysis population (SAP).

Variables (*n*, %)	Overall population (*N* = 2829)
Patients with AEs by type and severity	
Any AE	488 (17.2)
≥ 1 SAE	29 (1.0)
≥ 1 ADR	129 (4.6)
≥ 1 serious ADR	1 (0.04)
Treatment discontinuation due to ≥ 1 AE	85 (3.0)
Study discontinuation due to ≥ 1 AE	28 (1.0)
Death due to ≥ 1 AE	0 (0)
Patients with AEs by SOC (≥ 1%)	
GI disorders	242 (8.6)
Infections and infestations	96 (3.4)
Nervous system disorders	60 (2.1)
Psychiatric disorders	52 (1.8)
Respiratory, thoracic, and mediastinal disorders	43 (1.5)
Musculoskeletal and connective tissue disorders	39 (1.4)
General disorders and administration site conditions	37 (1.3)
Skin and subcutaneous tissue disorders	29 (1.0)

Abbreviations: GI, gastrointestinal; SOC, system organ class.

In addition, 129 (4.6%) of the 2829 patients in the SAP reported at least one ADR, with most of the ADRs being mild (*n* = 114, 4.0%) or moderate (*n* = 17, 0.6%). The most common ADRs were GI disorders, which were reported by 83 (2.9%) patients in the SAP, all of which were mild or moderate, with constipation and nausea being the most common (18 [0.6%] for each). The proportion of patients in the SAP with ADRs by system organ class or preferred term is shown in Table S4. No severe ADRs related to the GI system were reported, while one case of pneumonia was reported as a severe ADR (0.04%).

In addition, 85 (3.0%) patients discontinued treatment because of AE, and 28 (1.0%) patients discontinued the study because of AE. No deaths were reported during the study period.

### 3.3 Typical GERD Symptoms in the EAPRE

The EAPRE included 1796 patients with RE diagnosed by investigators in real‐world clinical practice (Figure 1). Of the 1040 out of these 1796 patients who had undergone endoscopy at baseline, 935 (89.9%) were classified as LA grade A/B (LA grade A, *n* = 553; LA grade B, *n* = 382) and 105 (10.1%) as LA grade C/D (LA grade C, *n* = 79; LA grade D, *n* = 26).

**FIGURE 2:**
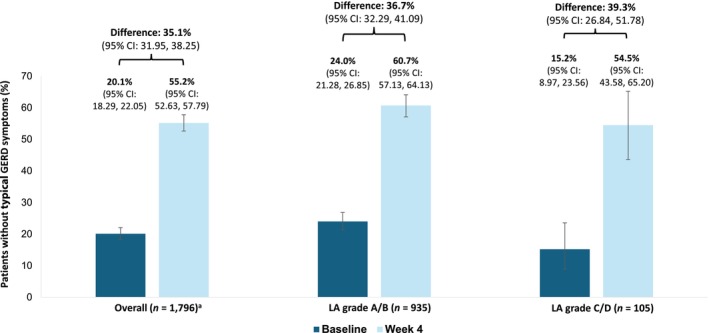
Patients without typical gastroesophageal reflux disease (GERD) symptoms at baseline and Week 4 in the effectiveness analysis population with reflux esophagitis (EAPRE) and differences in the proportions of patients without typical GERD symptoms between the baseline and Week 4 in the EAPRE, including those with Los Angeles (LA) grade A/B or C/D. ^†^Patients in the EAPRE (*n* = 1796) who were diagnosed clinically and defined as those in the safety analysis population (SAP) who had ≥ 1 effectiveness endpoint assessment at Clinical Visit 2 (Week 4 ± 2) and/or Clinical Visit 4 (Week 8 ± 2), and with Medical Dictionary for Regulatory Activities (MedDRA) Lowest Level Term (LLTs) coded as “reflux esophagitis,” “erosive esophagitis,” “reflux oesophagitis,” “erosive oesophagitis,” or “ongoing.” Abbreviation: CI, confidence interval.

The proportion of patients in the EAPRE (*n* = 1796) without typical GERD symptoms increased by 35.1% (95% CI 31.95–38.25) at Week 4 compared with baseline (from 20.1% at baseline to 55.2% at Week 4). Similar symptomatic improvement was observed in the subgroup of patients with LA grade A/B or C/D (Figure 2).

### 3.4 Symptom Relief From Heartburn and Regurgitation, and Improvement of GERDQ Score

A total of 1330 patients in the EAPRE reported heartburn or regurgitation symptom at baseline. For those who experienced heartburn during the day or night at baseline, the proportions of patients with complete symptom relief throughout all of the day time or nighttime in the first week of treatment were 32.3% (314 of 971) and 43.8% (427 of 975), respectively; while in the first 2 weeks of treatment, the respective percentages were 58.0% (572 of 986) and 68.9% (681 of 988). The same trend was observed in the subgroup of patients with LA grade A/B, and to a numerically greater extent by those with LA grade C/D (Table 3).

**TABLE 3:** Proportions of patients who achieved complete symptom relief of heartburn, nighttime heartburn, regurgitation, and nighttime regurgitation at baseline in the first week and the first 2 weeks of treatment in the effectiveness analysis population with reflux esophagitis (EAPRE), including those with Los Angeles (LA) grade A/B or C/D.

Complete symptom relief	Statistics	First week^a^, ^b^			First 2 weeks^b^, ^c^		
		Overall (*n* = 1796)^d^	LA grade		Overall (*n* = 1796)^d^	LA grade	
			A/B (*n* = 935)	C/D (*n* = 105)		A/B (*n* = 935)	C/D (*n* = 105)
Heartburn^e^	% (*n*/*N*)	32.3 (314/971)	33.7 (163/483)	44.9 (31/69)	58.0 (572/986)	59.9 (294/491)	66.7 (46/69)
	95% CI	29.40–35.38	29.54–38.16	32.92–57.38	54.86–61.12	55.39–64.24	54.29–77.56
Nighttime heartburn	% (*n*/*N*)	43.8 (427/975)	46.5 (225/484)	52.2 (36/69)	68.9 (681/988)	70.5 (347/492)	76.8 (53/69)
	95% CI	40.65–46.98	41.97–51.04	39.80–64.35	65.94–71.80	66.28–74.52	65.09–86.13
Regurgitation	% (*n*/*N*)	27.0 (262/971)	28.0 (136/485)	39.7 (27/68)	50.8 (501/987)	50.8 (250/492)	63.2 (43/68)
	95% CI	24.21–29.89	24.08–32.27	28.03–52.30	47.59–53.92	46.30–55.32	50.67–74.61
Nighttime regurgitation	% (*n*/*N*)	40.5 (395/976)	43.1 (210/487)	47.1 (32/68)	63.0 (622/987)	63.8 (314/492)	73.5 (50/68)
	95% CI	37.37–43.63	38.67–47.65	34.83–59.55	59.92–66.04	59.40–68.07	61.43–83.50

Abbreviation: CI, confidence interval.

^a^ Complete symptom relief of heartburn or regurgitation at the first week is defined as patients without heartburn or regurgitation (score 0) from Day 1 to Day 7.

^b^ *n* indicates patients without heartburn or regurgitation for all of the day/nighttime heartburn or regurgitation; *N*, for symptom relief during the first week or the first 2 weeks. Patients with at least one missing day of this data were not included in the denominator; for symptom relief during the first 2 weeks, patients without ≥ 7 consecutive days of data were not included in the denominator.

^c^ Complete symptom relief of heartburn or regurgitation in the first 2 weeks were defined as patients without heartburn or regurgitation (score 0) for ≥ 7 consecutive days between Day 1 and Day 14.

^d^ Patients in the EAPRE (*n* = 1796) were diagnosed clinically and defined as those in the safety analysis population (SAP) who had ≥ 1 effectiveness endpoint assessment at Clinical Visit 2 (Week 4 ± 2) and/or Clinical Visit 4 (Week 8 ± 2), and with Medical Dictionary for Regulatory Activities (MedDRA) Lowest Level Term (LLTs) coded as “reflux esophagitis,” “erosive esophagitis,” “reflux oesophagitis,” “erosive oesophagitis,” or “ongoing.”

^e^ For all the day time.

For patients who experienced regurgitation during the day time or nighttime at baseline, the percentages of those with complete symptom relief throughout all of the day time or nighttime in the first week of treatment were 27.0% (262 of 971) and 40.5% (395 of 976), respectively; while in the first 2 weeks of treatment, the respective percentages were 50.8% (501 of 987) and 63.0% (622 of 987). The same trend was observed in the subgroup of patients with LA grade A/B, and to a numerically greater extent by those with LA grade C/D (Table 3).

The mean GERDQ score decreased by −1.70 ± 2.80 (95% CI −1.80 to −1.51; *n* = 1464) from 8.40 ± 2.72 (*n* = 1794) at baseline to 6.80 ± 1.79 (*n* = 1465) at Week 4, where negative values represented improvement. Similar results were observed in the subgroup of patients with LA grade A/B or C/D (Table S5).

### 3.5 Endoscopic Assessment in EAPRE

The number of patients who underwent follow‐up endoscopic assessment at Week 4 was 32 (1.8%) and that at Week 8 was 8 (0.4%). Therefore, the cumulative number of patients at endoscopic assessment at Week 8 (± 2 weeks) was 40 (2.2%). Of these patients, 23 of 32 (71.9%, 95% CI 53.3–86.3) showed endoscopic healing by Week 4 (±2 weeks), and cumulatively 26 of 40 (65.0%, 95% CI 48.3–79.4) showed endoscopic healing by Week 8 (±2 weeks).

### 3.6 QoL Assessment

Of the patients in the EAPRE with available data (*n* = 1360), the mean global score change in the PSQI from baseline to Week 4 was −0.70 ± 2.31 (95% CI −0.79 to −0.55), where negative values represented improvement (Table 4). Similar results were observed in the subgroup of patients with LA grade A/B or C/D (Table 4).

**TABLE 4:** Changes from baseline to Week 4 in Pittsburgh Sleep Quality Index (PSQI) in the effectiveness analysis population with reflux esophagitis (EAPRE), including patients with Los Angeles (LA) grade A/B or C/D.

PSQI global score	Statistics	Overall EAPRE (*N* = 1796)^a^	LA grade	
			A/B (*n* = 935)	C/D (*n* = 105)
Baseline	Patients (*n*)	1720	905	101
	Mean ± SD	5.60 ± 3.38	5.40 ± 3.38	5.60 ± 3.56
Week 4	Patients (*n*)	1412	747	83
	Mean ± SD	4.90 ± 3.06	4.80 ± 3.13	4.90 ± 2.96
Change from baseline to Week 4	Patients (*n*)	1360	728	80
	Mean ± SD	−0.70 ± 2.31	−0.70 ± 2.28	−0.80 ± 2.63
	95% CI	−0.79 to −0.55	−0.84 to −0.51	−1.34 to −0.16

Abbreviations: CI, confidence interval; SD, standard deviation.

^a^ Patients in the EAPRE (*n* = 1796) are diagnosed clinically and defined as those in the safety analysis population (SAP) who have ≥ 1 effectiveness endpoint assessment at Clinical Visit 2 (Week 4 ± 2) and/or Clinical Visit 4 (Week 8 ± 2), and with Medical Dictionary for Regulatory Activities (MedDRA) Lowest Level Term (LLTs) coded as “reflux esophagitis,” “erosive esophagitis,” “reflux oesophagitis,” “erosive oesophagitis,” or “ongoing.”

In patients with available EQ‐5D‐5L index data, the mean score at baseline was 0.93 ± 0.09 (*n* = 1777) and that at Week 4 was 0.96 ± 0.07 (*n* = 1456), with a change of 0.03 ± 0.08 (95% CI 0.0251–0.0335; *n* = 1443), where positive values represented improvement. For the dimension of pain or discomfort, the mean score at baseline was 1.70 ± 0.75 (*n* = 1785), and 1.40 ± 0.59 (*n* = 1459) at Week 4, with a change of −0.30 ± 0.77 (95% CI −0.34 to −0.26; *n* = 1453), where negative values represented improvement. For the dimension of anxiety or depression, the mean score at baseline was 1.40 ± 0.63 (*n* = 1785) and at Week 4 it was 1.30 ± 0.51 (*n* = 1457), with a change of −0.20 ± 0.63 (95% CI −0.20 to −0.13; *n* = 1449), where negative values represented improvement.

In patients with available EQ‐VAS data, the mean score at baseline was 81.3 ± 12.8 (*n* = 1792) and at Week 4 it was 85.7 ± 10.6 (*n* = 1456), with a change of 4.50 ± 11.28 (95% CI 3.87–5.03; *n* = 1454), with positive values representing improvement. Similar results for change from baseline to Week 4 in mean EQ‐5D‐5L index score and EQ‐VAS score were observed in patients with LA grade A/B or C/D.

## 4 Discussion

To our knowledge, VIEW is so far the largest prospective, real‐world study to evaluate the safety of vonoprazan in patients and effectiveness in those with RE. Our results showed that a low proportion of patients receiving vonoprazan experienced at least one AE (17.2%), SAE (1.0%), ADR (4.6%), and treatment discontinuation due to AE (3.0%). These results are generally consistent with previous studies in Asia, the United States, or Europe regarding the incidence of AEs (30.2%) or treatment‐emergent AEs (TEAEs) (22.2%–38.1%), SAEs (0.6%) or serious TEAEs (0%–1.2%), with GI disorders being the most common AE or TEAE [11, 13, 38]. The results of VIEW therefore support the short‐term (≤ 8 weeks) tolerability of vonoprazan in real‐world clinical practice in China.

The results for symptom diaries indicate that patients with RE receiving vonoprazan experienced rapid and complete symptom relief within 1–2 weeks of administration, with improvements (when compared with baseline) in complete heartburn and regurgitation relief during all of the day and night. In subgroup analyses, symptom relief of heartburn and regurgitation (all day time and nighttime) was also observed in patients in the LA grade A/B subgroup to a similar extent as the overall patient population, and to a numerically greater extent in the LA grade C/D subgroup. First, we consider the rapid and complete symptom relief observed in patients during the first week of VIEW notable, given recent calls for treatments that provide a rapid reduction in the burden of symptoms experienced by patients with RE [39]. These results are also consistent with reports of patients with non‐erosive reflux disease experiencing symptom relief from heartburn within 24 h of vonoprazan administration [40, 41]. Second, the improvement in patients with nighttime symptoms in VIEW indicates that vonoprazan may benefit those with nocturnal acid breakthrough, which is defined as intragastric pH < 4 at nighttime for at least 60 min [42]. Third, similar to the symptom improvement observed in the subgroup of patients with LA grade C/D in VIEW, another study reported an improvement in endoscopic healing through 8 weeks in those with LA grade C/D who received vonoprazan over those who received lansoprazole [11]. Taken together, these findings indicate that vonoprazan provides rapid and complete relief from heartburn and regurgitation (all day and night) within 1–2 weeks of administration, improves the QoL of patients (particularly sleeping), and may also offer particular benefit to patients with LA grade C/D RE.

The results from VIEW are the first from a large sample of patients with RE in China to indicate the benefit of vonoprazan on QoL. Regarding the quality of sleep in patients with RE who received vonoprazan, the change in the PSQI score indicated moderate improvement in sleep quality from baseline to Week 4 (−0.7, 95% CI −0.8 to −0.6). This improvement may be partly due to the relief from nighttime heartburn and regurgitation. Moreover, the differences in EQ‐5D‐5L index score and EQ‐VAS score also indicate an improvement in the QoL of patients with RE (irrespective of severity) after treated with vonoprazan, particularly in the dimensions of pain/discomfort and anxiety/depression. Further investigation is needed to address the sparse reporting on symptom improvement with vonoprazan on the QoL of patients in Asia.

The present study includes several limitations when interpreting results. First, the single‐arm, non‐interventional, open‐label design without a control group precludes causal inferences regarding safety and effectiveness. Observed improvements may therefore reflect the natural course of disease, placebo effects, or concomitant treatments. While outside the scope of the present study, future investigations may consider including a PPI control group or data from the same hospital(s) on patients with RE who received PPI therapy during the same or similar period to construct a concurrent or historical control cohort. Second, as there was no control or comparison group in this real‐world study, alternative explanations for the observed changes from baseline cannot be ruled out [43, 44, 45, 46, 47]. A higher rate of lost‐to‐follow‐up might also have been observed in our study when compared with that of clinical trials. Placebo‐controlled studies with appropriate statistical analyses (e.g., propensity score matching and multivariate adjustment) are also required to confirm improvements in QoL in patients who receive vonoprazan, including correlations between changes in QoL or sleep scores and GERD symptoms. Third, there was a potential for bias, as the study was open‐label and awareness of treatment might have influenced the outcomes. Fourth, regarding safety, co‐primary endpoints did not include hierarchical ranking; only ADRs were related to vonoprazan, and there were no assessments of potential confounders (e.g., extensive concomitant medication use). Fifth, regarding treatment efficacy, there was a low rate of re‐evaluation via endoscopy, with only 40 of the 1796 patients in the EAPRE undergoing endoscopy at Week 4 and/or Week 8 as suggested by the protocol, which might have affected the accuracy of endoscopy‐related results. In addition, symptom improvement was assessed as a secondary outcome via self‐reported patient diaries, which introduces potential recall and reporting biases.

In conclusion, VIEW provides the largest real‐world study of vonoprazan and supports the short‐term (≤ 8 weeks) tolerability of vonoprazan, as well as symptom improvement in patients with RE in clinical practice in China.

## Funding

This study was funded by Takeda Pharmaceutical Company Limited, China.

## Conflicts of Interest

Min Hu Chen is the Co‐Editor‐in‐Chief for *Journal of Digestive Diseases* but was not involved in the editorial review or the decision to publish this article. Ying Lian Xiao, Hui Yang, Yi Xia Lu, Li Yang, Guo Xin Zhang, and Guo Liang Ye declare no conflicts of interest. Qi Song and Li Xie are employees of Takeda (China) International Trade Co. Ltd., and hold stock options in the company. Min Hu Chen has received speaker honoraria from Takeda China, AstraZeneca China, Xian Janssen, and Eisai China.

## Supporting information


**Table S1:** List of participating institutions.
**Table S2:** Definitions of complete symptom relief for the first and first 2 weeks.
**Table S3:** Patients who received prior or concomitant medications in the safety analysis population (SAP)^†^.
**Table S4:** Patients with adverse drug reactions (ADRs) by system organ class/preferred term (SOC/PT) in the safety analysis population (SAP).
**Table S5:** Changes from baseline to Week 4 in gastroesophageal reflux disease questionnaire (GERDQ) score in the effectiveness analysis population with reflux esophagitis (EAPRE) including patients with Los Angeles (LA) grade A/B or C/D.
**Figure S1:** Study design. ^a^Safety analysis population (SAP) (*n* = 2829). ^b^Effectiveness analysis population with reflux esophagitis (EAPRE) (*n* = 1796). Patients with sufficient benefit after 4 weeks by investigator evaluation then received a 2‐week safety follow‐up (i.e., to Week 6). While those with insufficient benefit after 4 weeks received vonoprazan for another 4 weeks (i.e., to Week 8), followed by a 2‐week safety follow‐up (i.e., to Week 10).

## Data Availability

The datasets, including the redacted study protocol, redacted statistical analysis plan, and redacted individual patient data supporting the results reported in this article, will be made available within 3 months of initial requests to researchers who provide a methodologically sound proposal. The data will be provided after de‐identification, in compliance with applicable privacy laws, data protection, and requirements for consent and anonymization. Proposals should be directed to Qi Song (qi.song@takeda.com). Data requestors will need to sign and abide by a data access agreement.
