# Supplementary material for: Real‐World Safety of Vonoprazan in Patients and Symptom Outcomes in Patients With Reflux Esophagitis in China: A Prospective, Non‐Interventional Study (VIEW)
Source: J Dig Dis. 2026 May 21;27(3-4):150–9. doi: 10.1111/1751-2980.70051 (PMC13281756; doi:10.1111/1751-2980.70051)
Supplement: Supplementary file 1 — Table S1: List of participating institutions. Table S2: Definitions of complete symptom relief for the first and first 2 weeks. Table S3: Patients who received prior or concomitant medications in the safety analysis population (SAP)†. Table S4: Patients with adverse drug reactions (ADRs) by system organ class/preferred term (SOC/PT) in the safety analysis population (SAP). Table S5: Changes from baseline to Week 4 in gastroesophageal reflux disease questionnaire (GERDQ) score in the effectiveness analysis population with reflux esophagitis (EAPRE) including patients with Los Angeles (LA) grade A/B or C/D. Figure S1: Study design. aSafety analysis population (SAP) (n = 2829). bEffectiveness analysis population with reflux esophagitis (EAPRE) (n = 1796). Patients with sufficient benefit after 4 weeks by investigator evaluation then received a 2‐week safety follow‐up (i.e., to Week 6). While those with insufficient benefit after 4 weeks received vonoprazan for another 4 weeks (i.e., to Week 8), followed by a 2‐week safety follow‐up (i.e., to Week 10). [file CDD-27-150-s001.docx]

**Supplementary Information**

1. **Approved Indications of Vonoprazan**

Vonoprazan has been approved in Japan for the treatment of gastric ulcer, duodenal ulcer, or reflux esophagitis (RE); prevention of recurrent gastric or duodenal ulcer associated with the administration of low-dose aspirin or non-steroidal anti-inflammatory drugs; and as an adjunct therapy for the eradication of *Helicobacter pylori* infection (gastric or duodenal ulcer, gastric mucosa-associated lymphatic tissue lymphoma, idiopathic thrombocytopenic purpura, post-endoscopic resection of early gastric cancer, or *H. pylori* gastritis) [1]. In the United States, vonoprazan has been approved for the healing and maintenance of healing of all grades of RE in adults, and in combination with amoxicillin alone or with amoxicillin and clarithromycin for *H. pylori* eradication in adults [2].

**References**

1. Evaluation and Licensing Division, Pharmaceutical and Food Safety Bureau Ministry of Health, Labour and Welfare, “Report on the Deliberation Results [November 26, 2014],” Available: https://www.pmda.go.jp/files/000211075.pdf.
2. U.S. Food and Drug Administration, “Vonoprazan [Revised November, 2023],” Accessed: October 17, 2024. Available from: https://www.accessdata.fda.gov/drugsatfda_docs/label/2023/215151s000lbl.pdf.
3. **Study Design and Patient Enrollment**

Patient-level data were collected, including patient medical records and patient-reported outcomes (patient diaries and questionnaires). The site investigator was responsible for ensuring that all the required data were collected per-protocol requirements. Any data provided by the patients served as the source document and were kept in study files by each site. Visits in this study were not mandatory, and clinical data were available only when collected under routine clinical practice.

**3. Statistical Analysis**

For categorical variables, descriptive statistics were conducted for the number and proportion of patients without missing values (the proportion was calculated after excluding the number of patients with missing values in the denominator). For numerical variables, descriptive statistics were conducted for the numbers of patients without and with missing values, and non-missing records were used to derive mean, standard deviation (SD), median, minimum and maximum.

Table S1. List of participating institutions.

| **Nos.** | **Participating institutions** |
| --- | --- |
| 1 | The First Affiliated Hospital of Sun Yat-sen University (Guangzhou, Guangdong Province, China) |
| 2 | Beijing Hospital (Beijing, China) |
| 3 | The First Hospital of China Medical University (Shenyang, Liaoning Province, China) |
| 4 | Heilongjiang Provincial Hospital (Harbin, Heilongjiang Province, China) |
| 5 | The First Hospital of Jilin University (Changchun, Jilin Province, China) |
| 6 | Jiangsu Province Hospital (Nanjing, Jiangsu Province, China) |
| 7 | Zhejiang Provincial People’s Hospital (Hangzhou, Zhejiang Province, China) |
| 8 | West China Hospital, Sichuan University (Chengdu, Sichuan Province, China) |
| 9 | The Second Affiliated Hospital and Yuying Children’s Hospital of Wenzhou Medical College (Wenzhou, Zhejiang Province, China) |
| 10 | Nanfang Hospital, Southern Medical University (Guangzhou, Guangdong Province, China) |
| 11 | The First Affiliated Hospital of Ningbo University (Ningbo, Zhejiang Province, China) |
| 12 | Shandong Provincial Hospital Affiliated to Shandong First Medical University (Jinan, Shandong Province, China) |
| 13 | Union Hospital, Tongji Medical College, Huazhong University of Science and Technology (Wuhan, Hubei Province, China) |
| 14 | The Second Affiliated Hospital of Guangzhou Medical University (Guangzhou, Guangdong Province, China) |
| 15 | General Hospital of Tianjin Medical University (Tianjin, China) |
| 16 | Nanjing Drum Tower Hospital, The Affiliated Hospital of Nanjing University Medical School (Nanjing, Jiangsu Province, China) |
| 17 | The Second Affiliated Hospital, Zhejiang University School of Medicine (Hangzhou, Zhejiang Province, China) |
| 18 | The Fourth Affiliated Hospital, Zhejiang University School of Medicine (Yiwu, Zhejiang Province, China) |
| 19 | Zhuhai People’s Hospital Medical Group (Zhuhai, Guangdong Province, China) |
| 20 | The Second Hospital & Clinical Medical School, Lanzhou University (Lanzhou, Gansu Province, China) |
| 21 | The First Affiliated Hospital of Nanchang University (Nanchang, Jiangxi Province, China) |
| 22 | Huazhong University of Science and Technology Union Shenzhen Hospital (Shenzhen Nanshan People’s Hospital) (Shenzhen, Guangdong Province, China) |
| 23 | The Second Affiliated Hospital of Nanjing Medical University (Nanjing, Jiangsu Province, China) |
| 24 | The Affiliated Yongchuan Hospital of Chongqing Medical University (Chongqing, China) |
| 25 | Beijing Tsinghua Changgung Hospital (Beijing, China) |
| 26 | The Third People’s Hospital of Chengdu (Chengdu, Sichuan Province, China) |
| 27 | Nanjing First Hospital (Nanjing, Jiangsu Province, China) |
| 28 | Wenzhou Central Hospital Affiliated to Wenzhou Medical University (Wenzhou, Zhejiang Province, China) |
| 29 | Xinhua Hospital Affiliated to Shanghai Jiao Tong University School of Medicine (Shanghai, China) |
| 30 | Qilu Hospital of Shandong University (Qingdao) (Qingdao, Shandong Province, China) |
| 31 | Tongji Hospital, Tongji Medical College, Huazhong University of Science and Technology (Wuhan, Hubei Province, China) |
| 32 | Weifang People’s Hospital (Weifang, Shandong Province, China) |
| 33 | Peking Union Medical College Hospital (Beijing, China) |
| 34 | Jiangxi Provincial People's Hospital (Nanchang, Jiangxi Province, China) |
| 35 | Henan Provincial People’s Hospital (Zhengzhou, Henan Province, China) |
| 36 | Yantai Affiliated Hospital of Binzhou Medical College (Yantai, Shandong Province, China) |
| 37 | The Affiliated Hospital of Hangzhou Normal University (Hangzhou, Zhejiang Province, China) |
| 38 | Changshu No. 2 People's Hospital (Changshu, Jiangsu Province, China) |
| 39 | Qilu Hospital of Shandong University (Jinan, Shandong Province, China) |

Table S2. Definitions of complete symptom relief for the first and first two weeks.

| **Symptoms** | **Definitions** |
| --- | --- |
| Complete symptom relief of heartburn during the first week | Patients without daytime heartburn (score of 0) and night-time heartburn (score of 0) from Day 1 to Day 7^†^ |
| Complete symptom relief of night-time heartburn during the first week | Patients without night-time heartburn (score of 0) from Day 1 to Day 7^†^ |
| Complete symptom relief of heartburn during the first two weeks | Patients without daytime heartburn (score of 0) and night-time heartburn (score of 0) for 7 or more consecutive days between Day 1 and Day 14^‡^ |
| Complete symptom relief of night-time heartburn during the first two weeks | Patients without night-time heartburn (score of 0) for 7 or more consecutive days between Day 1 and Day 14^‡^ |
| Complete symptom relief of regurgitation during the first week | Patients without daytime regurgitation (score of 0) and night-time regurgitation (score of 0) during daytime and night-time from Day 1 to Day 7^†^ |
| Complete symptom relief of night-time regurgitation during the first week | Patients without night-time regurgitation (score of 0) from Day 1 to Day 7^†^ |
| Complete symptom relief of regurgitation during the first two weeks | Patients without daytime regurgitation (score of 0) and night-time regurgitation (score of 0) for 7 or more consecutive days between Day 1 and Day 14^‡^ |
| Complete symptom relief of night-time regurgitation during the first two weeks | Patients without night-time regurgitation (score of 0) for 7 or more consecutive days between Day 1 and Day 14^‡^ |

^†^Patients with ≥ 1 missing day of data were not included in this analysis as well as the denominator when a percentage was provided.

^‡^Patients with any 7 consecutive days of data were included as well as the denominator when a percentage was provided. Symptoms were evaluated based on the patient’s symptom diary. The severity of daytime or night-time symptoms was measured with a 4-point scale: 0, none; 1, mild; 2, moderate; and 3, severe.

Table S3. Patients who received prior or concomitant medications in the safety analysis population (SAP)^†^.

| **Medications (*n*, %)** | **Prior medication^‡^** | **Concomitant medication^§^** | **Pooled^¶^** |
| --- | --- | --- | --- |
| Any medication | 248 (100) | 2258 (100) | 2294 (100) |
| Antacid | 44 (17.7) | 363 (16.1) | 391 (17.0) |
| Antidiarrheal microorganism | 27 (10.9) | 332 (14.7) | 346 (15.1) |
| β-lactam antibacterial, penicillin | 28 (11.3) | 319 (14.1) | 345 (15.0) |
| Digestive (including enzymes) | 26 (10.5) | 260 (11.5) | 278 (12.1) |
| Medication for functional gastrointestinal disorders | 16 (6.5) | 276 (12.2) | 287 (12.5) |
| Medication for peptic ulcer and GERD | 159 (64.1) | 1050 (46.5) | 1155 (50.3) |
| Unspecified herbal and traditional medicine | 32 (12.9) | 209 (9.3) | 233 (10.2) |
| Propulsive | 55 (22.2) | 825 (36.5) | 850 (37.1) |

^†^Only prior or concomitant medications at anatomical therapeutic chemical 3rd level received by > 10% of patients are included.

^‡^Prior medication is defined as the medication that stopped prior to the first dose of study medication.

^§^Concomitant medication is defined as the medication that ended on or after the date of first dose of study medication or was ongoing at study end.

^¶^Pooled is defined as patients with any medications. As a patient may have received both prior and concomitant medications, the sum of number in the “Prior medication” and “Concomitant medication” categories may exceed that in the “Pooled” column.

Abbreviation: GERD, gastroesophageal reflux disease.

Table S4. Patients with adverse drug reactions (ADRs) by system organ class/preferred term (SOC/PT) in the safety analysis population (SAP).

| **SOC/PT (*n*, %)** | **Patients (*n* = 1120)** |
| --- | --- |
| ≥ 1 ADR^†^ | 40 (3.6) |
| Gastrointestinal disorders | 27 (2.4) |
| Constipation | 7 (0.6) |
| Diarrhea | 7 (0.6) |
| Upper abdominal pain | 5 (0.4) |
| Abdominal pain | 4 (0.4) |
| Eructation | 3 (0.3) |
| Abdominal distension | 3 (0.3) |
| Dyspepsia | 2 (0.2) |
| Nausea | 2 (0.2) |
| Hyperchlorhydria | 1 (0.1) |
| Vomiting | 1 (0.1) |
| Nervous system disorders | 4 (0.4) |
| Headache | 2 (0.2) |
| Dizziness | 1 (0.1) |
| Tremor | 1 (0.1) |
| Psychiatric disorders | 3 (0.3) |
| Insomnia | 2 (0.2) |
| Abnormal dreams | 1 (0.1) |
| Skin and subcutaneous tissue disorders | 4 (0.4) |
| Pruritus | 1 (0.1) |
| Rash | 1 (0.1) |
| Urticaria | 1 (0.1) |
| Alopecia | 1 (0.1) |
| Musculoskeletal and connective tissue disorders | 2 (0.2) |
| Muscle twitching | 1 (0.1) |
| Pain in extremity | 1 (0.1) |
| Cardiac disorders | 1 (0.1) |
| Arrhythmia | 1 (0.1) |
| General disorders and administration site conditions | 1 (0.1) |
| Pyrexia | 1 (0.1) |
| Immune system disorders | 1 (0.1) |
| Anaphylactic reaction | 1 (0.1) |
| Respiratory, thoracic and mediastinal disorders | 1 (0.1) |
| Hiccups | 1 (0.1) |

^†^A patient could be included in more than one category.

Table S5. Changes from baseline to Week 4 in gastroesophageal reflux disease questionnaire (GERDQ) score in the effectiveness analysis population with reflux esophagitis (EAPRE) including patients with Los Angeles (LA) grade A/B or C/D.

| **GERDQ score** | **Statistics** | **Overall**  **(*n* = 1796)^†^** | **LA grade** | |
| --- | --- | --- | --- | --- |
|  |  |  | **A/B (*n* = 935)** | **C/D (*n* = 105)** |
| Baseline | Patients (*n*) | 1794 | 934 | 105 |
|  | Mean ± SD | 8.40 ± 2.72 | 8.20 ± 2.67 | 9.00 ± 2.82 |
| Week 4 | Patients (*n*) | 1465 | 773 | 88 |
|  | Mean ± SD | 6.80 ± 1.79 | 6.70 ± 1.77 | 7.00 ± 1.64 |
| Change from baseline to Week 4 | Patients (*n*) | 1464 | 773 | 88 |
|  | Mean ± SD | −1.70 ± 2.80 | −1.60 ± 2.72 | −2.10 ± 3.05 |
|  | 95% CI | −1.80 to −1.51 | −1.81 to −1.42 | −2.72 to −1.42 |

^†^Overall reflux esophagitis (*n* = 1796) was clinically diagnosed and defined as patients in the safety analysis population (SAP) who had ≥ 1 effectiveness end-point assessment at Clinical Visit 2 (Week 4 ± 2) and/or Clinical Visit 4 (Week 8 ± 2), and with Medical Dictionary for Regulatory Activities (MedDRA) Lowest Level Terms (LLTs) coded as “reflux esophagitis,” “erosive esophagitis,” “reflux oesophagitis,” “erosive oesophagitis,” or “ongoing.”

Abbreviations: CI, confidence interval; SD, standard deviation.

**Figure Legends**

**Figure S1**. Study design. ^a^Safety analysis population (SAP) (*n* = 2829). ^b^Effectiveness analysis population with reflux esophagitis (EAPRE) (*n* = 1796). Patients with sufficient benefit after 4 weeks by investigator evaluation then received a 2-week safety follow-up (i.e., to Week 6). While those with insufficient benefit after 4 weeks received vonoprazan for another 4 weeks (i.e., to Week 8), followed by a 2-week safety follow-up (i.e., to Week 10).
